# Association of Immune Thrombocytopenia and T-Lymphoblastic Lymphoma in a Pediatric Patient

**DOI:** 10.1155/2019/1425151

**Published:** 2019-12-17

**Authors:** Ryan A. Denu, Daniel R. Matson, Matthew J. Davis, Natalie J. Tedford, Christine E. Brichta, Carol A. Diamond, Margo L. Hoover-Regan

**Affiliations:** ^1^Medical Scientist Training Program, School of Medicine and Public Health, University of Wisconsin-Madison, Madison, WI, USA; ^2^Carbone Cancer Center, University of Wisconsin, Madison, WI, USA; ^3^Department of Pathology and Laboratory Medicine, School of Medicine and Public Health, University of Wisconsin-Madison, Madison, WI, USA; ^4^Department of Pediatrics, School of Medicine and Public Health, University of Wisconsin-Madison, Madison, WI, USA

## Abstract

Immune thrombocytopenia (ITP) is characterized by isolated thrombocytopenia of unclear etiology. We present a unique case of an 8-year-old girl with chronic ITP who was subsequently diagnosed with T-lymphoblastic lymphoma at age 11. The clinical course was complicated by the occurrence of nonepileptiform events with bizarre behavior changes following the administration of nelarabine and intrathecal and high-dose systemic methotrexate. This case highlights an unusual co-occurrence of hematologic malignancy and chronic ITP in an otherwise healthy child. We speculate that underlying genetic or immunologic lesions may predispose a subset of pediatric ITP patients to the development of hematologic malignancies.

## 1. Introduction

Immune thrombocytopenia (ITP) is a disorder characterized by isolated thrombocytopenia secondary to an aberrant antiplatelet antibody response. This results in a significantly shortened lifespan of circulating platelets with otherwise normal peripheral blood counts. The disease classically manifests with cutaneous and mucosal purpura; bone marrow examination typically shows increased megakaryocytes and normal numbers of other hematopoietic progenitors [1, 2]. Most cases of ITP are idiopathic and believed to reflect an autoimmune process secondary to perturbations in immune homeostasis (e.g., infections and autoimmune diseases) with resulting loss of peripheral tolerance [3].

Most children with ITP require only regular monitoring and will recover without treatment. However, treatment is indicated in a subset of patients with clinically significant disease, and this may include glucocorticoids, intravenous immunoglobulin, and anti-D immunoglobulin [4]. Treatment refractory cases may respond to rituximab and thrombopoietin receptor agonists (e.g., eltrombopag and romiplostim). Splenectomy is reserved for patients with chronic severe ITP who fail medical therapy [5]. Regardless of treatment regimen, about 10–30% will go on to develop chronic ITP, defined as thrombocytopenia greater than 12 months. [6–8] Risk factors for chronic ITP include older age, less severe thrombocytopenia at diagnosis, insidious onset of symptoms, and lack of a clear preceding/inciting event [7, 9, 10].

In adults, ITP is often associated with a concurrent hematological malignancy, most commonly chronic lymphocytic leukemia [11]. However, a formal relationship between hematologic malignancies and ITP has not been demonstrated in children. Herein, we report a rare case of chronic ITP that preceded the development of T-lymphoblastic lymphoma in a pediatric patient.

## 2. Case Presentation

**Figure 1:**
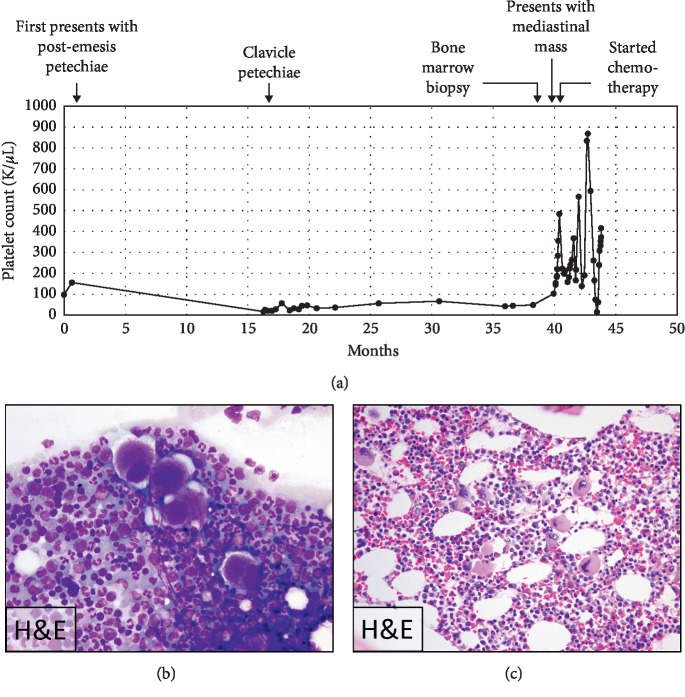
Trend of platelet count over time. (a) The patient's first platelet count was taken 37.1 months prior to the first point on the curve and was within normal limits at 292,000/*μ*L. (B-C) Bone marrow aspirate (b) and biopsy (c) demonstrating increased numbers of morphologically normal megakaryocytes.

An 8-year-old girl presented with bilateral, nonblanching facial petechiae following an episode of emesis. Her medical history was notable only for eczema. She was found to have thrombocytopenia with a platelet count of 96,000/*μ*L (reference 160,000–370,000/*μ*L). Three weeks later, a repeat complete blood count (CBC) demonstrated a borderline normal platelet count of 155,000/*μ*L. She then experienced a second episode of postemesis facial petechiae after which she underwent a 15-month period of observation. During this time, her platelet count remained between 30,000/*μ*L and 40,000/*μ*L. She then presented with petechiae on her trunk and extremities, and her platelet count was noted to be 14,000/*μ*L. She denied any bleeding episodes, including hematuria or hematochezia, but did complain of easy bruising on her extremities. No hepatosplenomegaly was noted on an otherwise normal physical examination; however, her parents felt she seemed more fatigued than her peers. Furthermore, they reported four years of intermittent febrile episodes associated with occasional syncope. Additional workup included an antinuclear antibody and erythrocyte sedimentation rate that were both within normal limits. A presumptive diagnosis of ITP was made. Because she had no active bleeding and her platelet count remained >10,000/*μ*L, she continued a period of observation only (Figure 1(a)).

Three years after her initial episode, she remained thrombocytopenic, and in consultation with her parents, she underwent bone marrow biopsy to rule out a bone marrow failure syndrome. This showed only linear and orderly trilineage hematopoiesis with increased numbers of morphologically normal megakaryocytes (Figures 1(b) and 1(c)).

**Figure 2:**
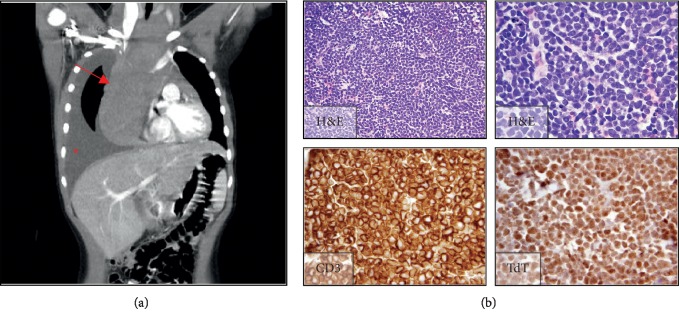
Imaging of mediastinal mass and pathology of lymph node biopsy sample. (a) Coronal view of CT scan of the chest, abdomen, and pelvis demonstrating right anterior mediastinal mass (arrow) measuring 9 × 6 × 18 cm and large right pleural effusion (asterisk). (b) Representative images showing sheets of large overtly malignant cells with blastic chromatin. Immunohistochemistry was strongly positive for CD3 and terminal deoxynucleotidyl transferase (TdT).

Two months later, she presented to her pediatrician's office with postprandial retrosternal and epigastric pain. She had been experiencing 1-2 weeks of poor appetite and intermittent vomiting with tender right cervical adenopathy and a four-pound weight loss. A chest X-ray demonstrated a right pleural effusion and bilateral hilar lymphadenopathy, at which point she was transferred to our hospital. On admission, her lactate dehydrogenase was elevated to 1302 U/L (ref 157–283 U/L), and uric acid was 12.7 mg/dL (ref 2.8–6 mg/dL). Platelets were 152,000/*μ*L, white blood cells were 7.5 K/*μ*L, and hemoglobin was 12.6 g/dL. CT of her neck, chest, abdomen, and pelvis demonstrated a right anterior mediastinal mass (9 × 6 × 18 cm) with extension through the thoracic inlet, encasement of the great vessels, and compression of the superior vena cava (Figure 2(a)). There was also diffuse thoracic and abdominal lymphadenopathy and bilateral renal infiltrates. Core biopsy of an enlarged lymph node showed medium-sized overtly malignant lymphoid cells with blastic chromatin, which were diffusely positive for TdT and CD3 by immunohistochemistry (Figure 2(b)). A diagnosis of T-lymphoblastic lymphoma was rendered. Concurrent flow cytometry of pleural fluid was remarkable for abundant medium- to large-size blasts, consistent with T-lymphoblastic lymphoma, whereas a bone marrow biopsy and lumbar puncture both showed no evidence of involvement by T lymphoblasts. These findings established a diagnosis of stage III T-ALL according to St. Jude staging criteria.

She was treated per Children's Oncology Group (COG) AALL1231, Arm A. She achieved a complete remission at the end of the induction phase of treatment. Nelarabine was added to the consolidation, delayed intensification, and maintenance phases, in light of this drug conferring benefit on a previous COG study for patients with T-cell acute lymphoblastic leukemia and T-lymphoblastic lymphoma, AALL 0434. Rasburicase and allopurinol were used to manage tumor lysis syndrome. Treatment was complicated by encephalopathy, which developed after nelarabine administration and later after intrathecal and high-dose systemic methotrexate administration. This was characterized by intermittent nonepileptiform events (assessed by electroencephalogram) lasting between 10 and 90 minutes. These events were quite bizarre; for example, our patient would speak in British or Creole accents, recite Disney quotes or songs repeatedly, or make animal noises. She was aware of the events and could sense their onset but seemingly had no control of what she said. These events were mitigated with the use of dextromethorphan rescue, which has previously been reported to abrogate methotrexate-induced neurotoxicity in pediatric patients [12].

The patient remains in remission and has continued to receive treatment per AALL1231. She is currently 15 months into treatment and has completed induction, consolidation, two cycles of interim maintenance, delayed intensification, and two cycles of maintenance chemotherapy. Her platelet count has increased since starting chemotherapy and is currently within normal limits (Figure 1).

## 3. Discussion

We report here an interesting association of chronic ITP with the development of T-lymphoblastic lymphoma in a child. In a literature search, we identified very few reported cases of concurrent ITP and T-cell malignancies in children and even fewer cases in which chronic ITP preceded the development of lymphoma or leukemia [13–16]. In one such case, a 13-month-old otherwise asymptomatic boy was found to be thrombocytopenic on routine blood work and 10 months later developed a large facial and periorbital hematoma after head trauma [15]. His platelets were reduced at 20,000/*μ*L and flow cytometry confirmed T-ALL with 63% circulating blasts. In another case, a 1-year-old boy was diagnosed with ITP, treated with low-dose prednisone, and then developed T-ALL three years later. His platelet counts returned to normal after completing chemotherapy [13].

Our reports and others raise the possibility that chronic ITP may serve as a predisposing factor for the development of hematologic malignancies in children, specifically acute lymphoblastic leukemias and lymphomas. This would not be entirely surprising, as ITP is associated with hematologic malignancies in adults. However, to date, such a relationship has not been formally demonstrated in the pediatric setting. Emerging data provide several possible mechanisms by which this could occur. First, there is growing evidence that ITP results in significant T-cell dysfunction. This includes a reduction in the number and function of T-regulatory cells and a concurrent increase in the number of T-helper cells [17–19]. These changes occur alongside increased levels of interferon *γ*, tumor necrosis factor *α*, and interleukin-2 and decreased levels of interleukin-4 [20]. These data suggest that T-cell-related proinflammatory cytokines are central to the pathogenesis of ITP and that children with ITP may have broadly dysregulated T-cell function.

We also consider whether a subset of children with ITP may harbor predisposing genetic factors, which could also put them at risk for the development of hematological malignancies. In such cases, the child's chronic ITP would in fact represent an undiagnosed inherited disorder of thrombocytopenia. There are known mutations that predispose to thrombocytopenia; ANKRD26, RUNX1, and ETV6-related thrombocytopenias are among several recently described entities that are speculated to predispose patients to hematologic malignancies [21]. ANKRD26 mutations are typically in the 5′ untranslated region (UTR) and prevent RUNX1 and FLI1 from binding and silencing ANKRD26, resulting in inappropriate ANKRD26 expression and increased signaling via the myeloproliferative leukemia (MPL) oncogenic pathway [22]. RUNX1 is a transcription factor required for hematopoietic stem cell differentiation, and loss-of-function mutations prevent differentiation into platelets and perpetuate a dedifferentiated state, leading to leukemic transformation [23]. ETV6 is a transcriptional repressor that is required for survival of hematopoietic stem cells and for promoting late phases of megakaryopoiesis [24]. Further work will be needed to assess these potential mechanisms.

Our case raises a question of whether or not children with chronic ITP are predisposed to lymphoblastic lymphomas/leukemias. Additional research into the immunology and genetics of children with chronic ITP should consider a propensity for the development of hematologic malignancies.

## Abbreviations

ITP: Immune thrombocytopenia
T-ALL: T-cell acute lymphoblastic leukemia/lymphoma.
